# Enitociclib, a Selective CDK9 Inhibitor, Induces Complete Regression of MYC+ Lymphoma by Downregulation of RNA Polymerase II Mediated Transcription

**DOI:** 10.1158/2767-9764.CRC-23-0219

**Published:** 2023-11-09

**Authors:** Melanie M. Frigault, Arushi Mithal, Harvey Wong, Beatrix Stelte-Ludwig, Vinay Mandava, Xin Huang, Joseph Birkett, Amy J. Johnson, Raquel Izumi, Ahmed Hamdy

**Affiliations:** 1 Vincerx Pharma, Inc., Palo Alto, California.; 2 Vincerx Pharma GmbH, Monheim am Rhein, Germany.

## Abstract

Double-hit diffuse large B-cell lymphoma (DH-DLBCL) is an aggressive, and often refractory, type of B-cell non–Hodgkin lymphoma (NHL) characterized by rearrangements in MYC and BCL2. Cyclin-dependent kinase 9 (CDK9) regulates transcriptional elongation and activation of transcription factors, including MYC, making it a potential targeted approach for the treatment of MYC+ lymphomas. Enitociclib is a well-tolerated and clinically active CDK9 inhibitor leading to complete metabolic remissions in 2 of 7 patients with DH-DLBCL treated with once weekly 30 mg intravenous administration. Herein, we investigate the pharmacodynamic effect of CDK9 inhibition in preclinical models and in blood samples from patients [DH-DLBCL (*n* = 10) and MYC+ NHL (*n* = 5)] treated with 30 mg i.v. once weekly enitociclib. Enitociclib shows significant regulation of RNA polymerase II Ser2 phosphorylation in a MYC-amplified SU-DHL-4 cell line and depletion of MYC and antiapoptosis protein MCL1 in SU-DHL-4 and MYC-overexpressing SU-DHL-10 cell lines *in vitro*. Tumor growth inhibition reaching 0.5% of control treated SU-DHL-10 xenografts is achieved *in vivo* and MYC and MCL1 depletion as well as evidence of apoptosis activation after enitociclib treatment is demonstrated. An unbiased analysis of the genes affected by CDK9 inhibition in both cell lines demonstrates that RNA polymerase II and transcription pathways are primarily affected and novel enitociclib targets such as PHF23 and TP53RK are discovered. These findings are recapitulated in blood samples from enitociclib-treated patients; while MYC downregulation is most robust with enitociclib treatment, other CDK9-regulated targets may be MYC independent delivering a transcriptional downregulation via RNA polymerase II.

**Significance:**

MYC+ lymphomas are refractory to standard of care and novel treatments that downregulate MYC are needed. The utility of enitociclib, a selective CDK9 inhibitor in this patient population, is demonstrated in preclinical models and patients. Enitociclib inhibits RNA polymerase II function conferring a transcriptional shift and depletion of MYC and MCL1. Enitociclib intermittent dosing downregulates transcription factors including MYC, providing a therapeutic window for durable responses in patients with MYC+ lymphoma.

## Introduction

Diffuse large B-cell lymphoma (DLBCL) is the most common non–Hodgkin lymphoma (NHL) subtype (1). Although standard immunochemotherapy often cures patients with DLBCL (2, 3), up to 40% of patients will eventually relapse (4, 5). Many of these patients are diagnosed with high-grade B-cell lymphoma with MYC and BCL2 rearrangement (HGBL-MYC/BCL2; refs. 6, 7) a subtype also known as double-hit diffuse large B-cell lymphoma (DH-DLBCL; refs. 8, 9). *MYC* and *BCL2* gene rearrangements result in dysregulated activation of translocation fusion proteins that drive oncogenic transcription and antiapoptotic signaling, respectively (10). Patients with DH-DLBCL have poor prognosis compared with patients with other DLBCL subtypes (11) and are generally refractory to standard-of-care chemotherapy (12).

Cyclin-dependent kinase 9 (CDK9) belongs to CDK family of proteins, which regulate crucial cell functions in a complex with specific cyclin subunits, including cell cycle or transcription control (13). CDK9 acts as a catalytic subunit that dimerizes with cyclin T subunit, forming the positive transcription elongation factor b (P-TEFb) complex (14). P-TEFb is identified as the key regulator of transcription by RNA polymerase II (15). CDK9 phosphorylates the carboxyl-terminal domain of RNA polymerase II at the serine 2 residue (p-Ser2; refs. 16, 17) and in turn is required for the elongation of mRNA transcripts. MYC is a pleiotropic transcription factor, which controls cell cycle, DNA damage repair, and cell metabolism by regulating numerous target genes (18). *MYC* mRNA has a short half-life; therefore, high rates of *MYC* transcription are necessary to drive oncogenic signaling (19–21). Myc protein expression is consequently dependent on CDK9 phosphorylation of p-Ser2. Mcl-1 is an antiapoptotic protein belonging to Bcl-2 family (22). Like Bcl-2, Mcl-1 promotes cell survival (23) and many hematologic malignancies are dependent on the antiapoptotic activity of Bcl-2 or Mcl1 (24). Mcl-1 has been implicated as an apoptotic regulator in Eµ-*Myc*–driven lymphomas and is regulated by P-TEFb (25). Dinaciclib, a pan-CDK inhibitor, has been shown to downregulate MCL1 leading to apoptosis induction and antitumor efficacy in DLBCL preclinical models (25) and clinical activity has been demonstrated in patients with relapsed/refractory chronic lymphocytic leukemia (CLL; ref. 26).

Enitociclib, formerly known as VIP152, is a well-tolerated and clinically active CDK9 inhibitor that has led to durable complete metabolic remission (CR) in 2 of 7 patients with DH-DLBCL treated once weekly with 30 mg enitociclib by intravenous infusions (27, 28). The pharmacodynamic effect of in blood of patients treated with enitociclib at dose levels lower than 30 mg demonstrates that *MYC*, *MCL1,* and *PCNA* mRNA downregulation is not as robust and/or durable (29). Enitociclib is administered intravenously once weekly to allow for on-target Mcl-1 downregulation in neutrophils to recover before the next dose providing a wider therapeutic window unlike other orally administered CDK9 inhibitors in development such as atuveciclib and KB-0742 (30, 31). Previous studies using an acute myeloid leukemia (AML) xenograft model have demonstrated excellent *in vivo* efficacy of enitociclib (32). Herein, we elucidate the *in vitro* mechanism of action of enitociclib, atuveciclib, and KB-0742 in MYC-driven DLBCL preclinical models and demonstrate that enitociclib treatment induces a depletion of *MYC* as well as describe novel CDK9 inhibitor target genes that are downregulated on a different time course to deliver an “oncogenic shock” (33, 34) in cells addicted to MYC expression. These findings are confirmed in the blood of patients with MYC+ lymphoma treated with enitociclib and elucidate the mechanism of action of P-TEFb targeting by selective CDK9 inhibition for the treatment of patients with NHL with MYC-driven disease.

## Methods and Materials

### Compounds and Cell Lines

Human DLBCL cell lines SU-DHL-4 (#ACC 495) and SU-DHL-10 (#ACC 576) were obtained from DSMZ (German Collection of Microorganisms and Cell Cultures). The supplier used PCR-based DNA profiling of polymorphic short tandem repeats for authentication and *Mycoplasma* testing prior to shipment. Cells were cultured in suspension at 37°C in a humidified atmosphere containing 5% CO_2_. CDK9 inhibitors KB-0742 (MedChemExpress, #HY-137478A), enitociclib, and atuveciclib (Vincerx Pharma, Inc.) were used. A subset of 35 lymphoma cell lines is from a 500-cell line panel where the effect of enitociclib on cell viability using CellTiter-Glo luminescent cell viability was determined (OmniScreen, CrownBioscience). Genetic status and gene expression level for *MYC* and *MCL1* was obtained from CrownBioscience.

### *In Vitro* Washout Experiments

Evaluation of the pharmacodynamic extent and duration of CDK9 inhibition were performed *in vitro* using the human DLBCL cell lines SU-DHL-4 and SU-DHL-10, harboring *MYC* amplification and *MYC* overexpression with P57T mutation, respectively. Cells were seeded 18 hours prior to treatment for 4 hours with one of three CDK9 inhibitors, enitociclib (0.25 and1 µmol/L), atuveciclib (1 µmol/L) or KB-0742 (1 µmol/L), cells were washed, and incubation continued until 48 hours posttreatment. Samples were collected before treatment (−4), immediately after treatment (0), and at 1, 2, 4, 8, 12, 16, 24, and 48 hours after washout for RNA sequencing (RNA-seq), qPCR, and Western blotting analyses.

### RNA-seq and qPCR

Total RNA was extracted from blood of patients and cell lines using RNeasy Plus Mini Kit (QIAGEN, #74136) and cDNA prepared with the Maxima First Strand cDNA Synthesis Kit for RT-PCR (Thermo Fisher Scientific, K1642) according to manufacturer's instructions. The integrity of purified total RNA was evaluated using an Agilent 2100 Bioanalyzer system and RNA 6000 Nano Kit (Agilent, #5067-1511). Differential gene expression analysis was performed (Fios Genomics) from RNA-seq data (Discovery Life Sciences). *MYC* and *MCL1* mRNA levels were measured by qPCR *in vitro* and *in vivo*, using a Taqman qPCR system (Thermo Fisher Scientific), TaqMan Fast Universal PCR Master Mix (2X) no AmpErase (#4352042) and TaqMan probes for *MYC* (Hs00153408_m1) and *MCL1* (Hs03043899_m1). RNA-seq data were analyzed using DESeq2 (35).

### Western Blotting

The levels of p-Ser2, p-Ser5 and total RNA polymerase II, Myc, Mcl-1, cleaved PARP (cPARP), and cleaved Caspase 3 were assessed by Western blotting and quantified near-infrared detection and background corrected quantification of fluorescence intensity on an Odyssey Imaging System with IRDye secondary antibodies (LI-COR Biosciences). For quantification, the protein levels were normalized to GAPDH or HSP90, expressed as a ratio to DMSO or vehicle control at matched timepoints. The normalized relative protein levels of p-Ser2 were used for a treatment effect comparison by performing an ANOVA using PROC MIXED in SAS. The ANOVA model included treatment, timepoints and their interactions as fixed effects. The least square means (LSM), differences between the LSMs and associated *P* values were generated. The following primary antibodies were used: Rpb1 NTD (D8L4Y) rabbit antibody [Cell Signaling Technology (CST), #14958], anti-RNA polymerase II subunit B1 (phospho CTD Ser-2, clone 3E10) rat antibody (Merck, 04-1571-I), recombinant anti-c-MYC rabbit antibody (Y69; Abcam, #ab32072), MCL1 (D5V5L) rabbit antibody (CST, #39224), cleaved-PARP (Asp214) (E2T4K) mouse antibody (CST, #32563), HSP90 (C45G5) rabbit antibody (CST, #4877), and GAPDH mouse antibody (Zytomed Systems, #ZYT-RGM2-6C5).

### Animal Experiments

A total of 10 × 10^6^ SU-DHL-10 cells were inoculated subcutaneously into 6–8 weeks old female C.B-17 SCID mice (Iffa Credo, Brussels). The mice were randomized to treatment groups at a tumor volume of 63–104 mm^3^ and treated (intravenously, once weekly) with 10 mg/kg enitociclib (60% PEG400, 10% ethanol, water), 15 mg/kg enitociclib (30% PEG400, 10% ethanol, water), or corresponding vehicle treatments. Mice were around 10–12 weeks of age at the start of study. All animal experiments were performed under the national animal welfare laws of Germany and approved by the local authorities.

Statistical analyses were performed using GraphPad Prism v9 (GraphPad Software). For tumor volumes in SU-DHL-10 xenograft model, statistics were performed using unpaired *t* test in comparison with the corresponding vehicle treatment. The obtained *P* values were adjusted for all analyses. Results were considered statistically significant when *P* < 0.05.

### Patients

Fifteen patients with refractory or relapsed DH-DLBCL or other MYC+ NHL were treated with enitociclib (30 mg, 21-day cycle, once weekly x3) as a 30-minute intravenous infusion on study days 1, 8, and 15 (NCT02635672). Longitudinal gene expression levels were analyzed by RNA-seq, and enitociclib plasma concentrations were measured using LC/MS-MS. The clinical study protocol was approved by the Institutional Review Board of participating institutions and complied with the Declaration of Helsinki, current Good Clinical Practice guidelines, and local laws and regulations. Written informed consent was provided by all participants prior to the initiation of any study-specific procedure.

### Pharmacokinetic and Pharmacodynamic Modeling of Patient Samples

All pharmacokinetic parameters were calculated by noncompartmental methods as described previously (36) using WinNonlin v5.3 (Certara LP). Pharmacokinetic/pharmacodynamic modeling was performed using SAAM II v2.3.2 (The Epsilon Group). Pooled pharmacokinetic data from all patients [*n* = 15 for single dose, cycle 1 day 1 (C1D1) and *n* = 11 for multiple doses, cycle 1 day 15 (C1D15)] were characterized using a one-compartment model with intravenous dosing. Subsequently, the pharmacokinetic/pharmacodynamic relationship of enitociclib plasma concentrations to *MCL1* and *MYC* mRNA change was characterized using an indirect response model (37) on pooled biomarker data where enitociclib concentrations inhibit the formation of *MCL1* or *MYC* mRNA as described by the following equation:



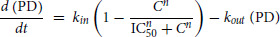



PD is defined as *MCL1* or *MYC* mRNA expression level as a percentage from baseline, *t* (h) is the time, *k_in_* (1/h) is the formation rate of *MCL1* or *MYC*, IC_50_ (µg/L) is the enitociclib concentration where there is 50% inhibition of PD, *k_out_* (h^−1^) is the rate constant describing the loss of PD*,* and *n* is the hill coefficient. At homeostasis, *k_in_ = k_out_* (PD); therefore, *k_out_* was replaced by *k_in_*/(PD)_initial_ where (PD)_initial_ is equal to 100%. Estimates of pharmacokinetic and pharmacodynamic parameters from pharmacokinetic/pharmacodynamic modeling of pooled data are presented as the estimate followed by CV in parentheses.

### Differential Gene Expression Analysis

RNA-seq data generated from the analysis of two cell lines treated with the three CDK9 inhibitors was analyzed to investigate the differences in gene expression levels between various posttreatment timepoints and the pretreatment samples. Quality control assessment and exploratory data analysis by principal component analysis (PCA) were done on raw counts and on normalized count data. Normalization of RNA-seq count data was done using trimmed mean of M-values normalization and transformed with voom, resulting in log_2_ counts per million with associated precision weights. To determine significantly differentially expressed genes (DEG) between different timepoints of treatment with DMSO (control), CDK9 inhibitor treatment and the pretreatment control samples, statistical comparisons were performed using linear modeling on normalized data as implemented in the Bioconductor package limma, and the final values were expressed as a percentage of baseline. Significance values (*P*_adj_) were adjusted for multiple testing, by controlling the FDR using the Benjamini–Hochberg procedure. Significant DEGs (at *P*_adj_ ≤ 0.05, fold change ≥2) identified were then analyzed for enrichment of Reactome pathways using a hypergeometric test, and the enrichment (*P* < 0.05) was assessed together for the upregulated and downregulated genes. RNA-seq data generated from blood samples taken from patients before and after their first dose (C1D1) or after multiple doses, before and after their third dose (C1D15) were analyzed and normalized using the same pipeline as cell lines for comparisons.

### Data Availability Statement

The data generated in this study are available in the National Center for Biotechnology Information Repository and Sequence Read Archive database with accession numbers: SAMN37546465 through SAMN37546748.

## Results

### Enitociclib Delivers Robust Inhibition of RNA Polymerase II Ser2 Phosphorylation for up to 48 Hours in Cell Lines

**FIGURE 1:**
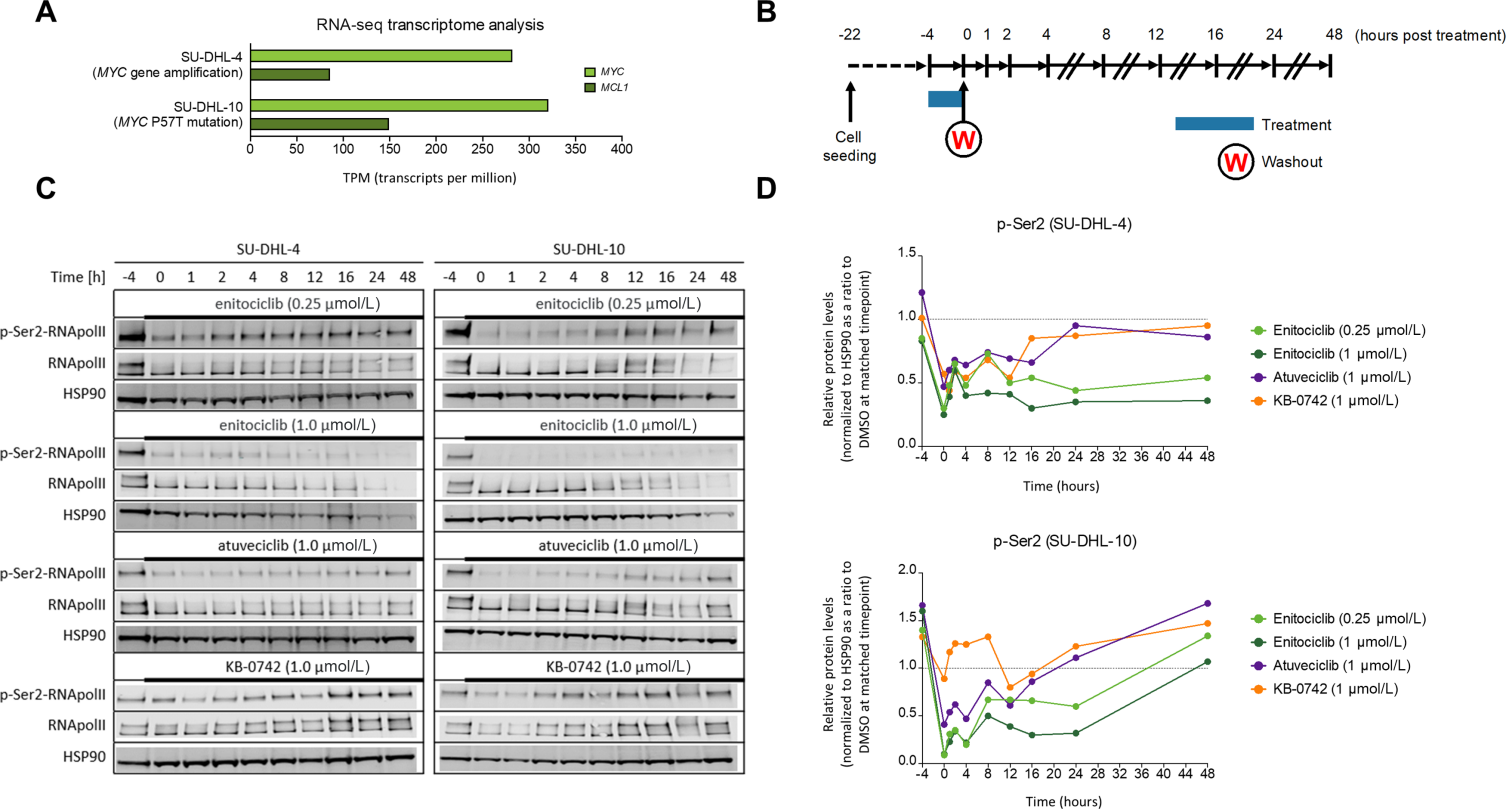
Enitociclib delivers robust inhibition of RNA polymerase II Ser2 phosphorylation for up to 48 hours in cell lines. **A,** The gene expression of *MYC,* and *MCL1* as determined by RNA-seq expressed as transcripts per million (TPM) from parental untreated SU-DHL-4 and SU-DHL-10 DLBCL cells. **B,** SU-DHL-4 and SU-DHL-10 DLBCL cells were seeded 18 hours prior to treatment with one of three CDK9 inhibitors: enitociclib (0.25 or 1 µmol/L), atuveciclib (1 µmol/L), or KB-0742 (1 µmol/L). Following the 4-hour treatment, the cells were washed, and the incubation continued for up to 48 hours. Samples were collected 4 hours before treatment (−4), immediately after treatment (0), and at 1, 2, 4, 8, 12, 16, 24, and 48 hours after washout for RNA-seq, qPCR, and Western blotting analysis. **C,** Western blots of phosphorylated Ser2 RNA polymerase II (p-Ser2-RNApolII), total RNApolII and HSP90 (**D**) quantification of p-Ser2-RNApolII protein levels (p-Ser2) in SU-DHL-4 and SU-DHL-10 DLBCL cells normalized to HSP90 as a ratio to DMSO matched timepoint.

Enitociclib induced cell cytotoxicity (IC_50_ values ranged from 0.043 to 0.152 µmol/L) in a panel of 35 human lymphoma cell lines. *MYC* and *MCL1* mRNA downregulation has been described previously from the blood of patients treated with enitociclib during dose escalation and across many cancer indications including NHL and solid tumors using a qPCR clinical trial assay (28, 29). Because these genes are thought to drive tumorigenesis, the genetic status and expression level of *MYC* and *MCL1* for each line is indicated (25); however, the correlations between these aberrations and enitociclib sensitivity and resistance were not possible as most lymphoma cell lines are sensitive to enitociclib treatment (Supplementary Table S1). Two MYC-driven DLBCL cell line models, SU-DHL-4 and SU-DHL-10, were used to evaluate the enitociclib mechanism of action with IC_50_ values of 43 nmol/L and 74 nmol/L, respectively. A 0.25 µmol/L concentration [unbound concentration = 167 nmol/L (250 nmol/L X 66.6% (fraction unbound in cell media))] was included in the *in vitro* study as it provided unbound concentrations comparable *C*_max_ [unbound *C*_max_ = 117 nmol/L (1,750 nmol/L (*C*_max_) X 6.65% (fu))] in patients dosed with 30 mg i.v. enitociclib once weekly. In patients with multiple dose data on C1D15 (*n* = 10), the unbound *C*_max_ range is 67.5 to 147 nmol/L. As we were investigating the effects of “oncogenic shock” induced by enitociclib, unbound *C*_max_ was deemed a relevant concentration to target *in vitro*. Furthermore, the mean t_1/2_ as measured in patients treated with multiple doses of enitociclib is 5.56 hours ± 1.93 hours and therefore a 4-hour pretreatment of 250 nmol/L enitociclib was used to model dosing *in vitro*. The 250 nmol/L *in vitro* enitociclib concentration used in these experiments is based on the clinical exposure of enitociclib in 15 patients with MYC+ lymphoma; however, this is an estimate and may be superphysiologic. Baseline genomic profiles of RNA-seq analysis demonstrated that both cell lines have comparable *MYC* transcript levels, despite different *MYC* DNA status where SU-DHL-4 cell line has *MYC* gene amplification and SU-DHL-10 has *MYC* P57T mutation driving *MYC* overexpression. The levels of *MCL1* mRNA in SU-DHL-10 cells were almost twice as high compared with the SU-DHL-4 cells (Fig. 1A). A 4-hour pretreatment with either enitociclib (0.25 or 1 µmol/L), atuveciclib (1 µmol/L), or KB-0742 (1 µmol/L) was washed out and the pharmacodynamic effect was observed at the indicated timepoints (Fig. 1B). In the SU-DHL-4 cell line, treatment with 0.25 µmol/L enitociclib decreased the levels of p-Ser2 by 50% for up to 48 hours. The extent of p-Ser2 inhibition was greater at the higher 1 µmol/L enitociclib treatment, sustaining for 48 hours. In contrast, treatment with 1 µmol/L KB-0742 elicited a 50% decrease for up to 12 hours in alignment with a previous report using a *MYC-*driven AML xenograft mouse model (31). Treatment with 1 µmol/L atuveciclib approached 50% inhibition of p-Ser2 for up to 16 hours (Fig. 1C and D), a marker known to be downregulated by atuveciclib in T-cell prolymphocytic leukemia models (38). In the SU-DHL-10 cell line, 1 µmol/L enitociclib was the only treatment able to achieve 50% reduction of p-Ser2 for up to 24 hours after treatment. Atuveciclib and KB-0742 also inhibited p-Ser2, but only in the presence of the treatment compound and not after washout (Fig. 1D). Comparison of each drug treatment demonstrates that the p-Ser2 changes on RNA polymerase II are statistically significant in the SU-DHL-4 cell line at all timepoints (Supplementary Table S2). The limitation of the experimental design is that the exposure of KB-0742 may not mimic the clinical pharmacokinetic profile. These are not public at time of writing therefore previously published concentration of 1 µmol/L was used in these studies (31).

### Enitociclib Depletes mRNA transcripts, *MYC* and *MCL1;* Downregulates Myc and Mcl1 Protein Levels and Activates Cell Death *In Vitro*

**FIGURE 2:**
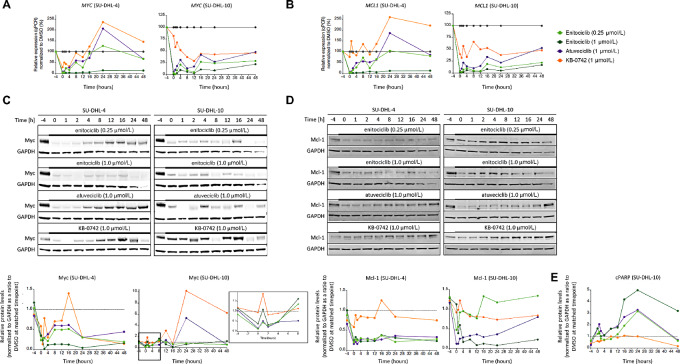
Enitociclib depletes mRNA transcripts, *MYC* and *MCL1;* downregulates Myc and Mcl-1 protein levels and activates cell death *in vitro MYC* (**A**) and *MCL1* (**B**) mRNA levels were determined using qPCR normalized to 18s rRNA from SU-DHL-10 and SU-DHL-4 DLBCL cells treated with indicated concentration of CDK9 inhibitor for 4 hours. The protein levels of Myc (**C**) and Mcl-1 (**D**) pretreatment and at indicated times after treatment washout were quantified relative to GAPDH in SU-DHL-10 and SU-DHL-4 cells. The inset shows Myc protein levels in SU-DHL-10 cells until 8 hours after treatment. **E,** The protein levels of cPARP is quantified from SU-DHL-10 DLBCL cells pretreatment and at indicated times after treatment.

Because CDK9 inhibition abrogates transcription elongation of mRNAs by RNA polymerase II, we next evaluated the extent and duration of the effect of CDK9 inhibition on mRNA levels of *MYC* and *MCL1* by qPCR. *MYC* and *MCL1* mRNA levels were decreased with all three CDK9 inhibitors initially; however, only atuveciclib and enitociclib were able to drive the depletion of *MYC* (Fig. 2A) and *MCL1* (Fig. 2B) mRNA transcript after treatment washout in SU-DHL-10 and SU-DHL-4 cell lines. In SU-DHL-10 cells, atuveciclib and KB-0742 were able to maintain approximately 50% depletion of the *MYC* and *MCL1* mRNA levels for up to 16 hours after treatment, whereas enitociclib inhibited *MYC* and *MCL1* mRNA levels approximately 75% for 16 to 48 hours after treatment. In *MYC*-amplified SU-DHL-4 cells, only 1 µmol/L enitociclib maintained durable depletion of *MYC* and *MCL1* mRNA levels over the 48-hour time course (Fig. 2A and B). At the protein level, 1 µmol/L enitociclib was the only treatment that drove sustained Myc (Fig. 2C) and Mcl-1 (Fig. 2D) protein depletion for 48 hours in the SU-DHL-10 cells; whereas this was achieved in SU-DHL-4 cells with 0.25 µmol/L enitociclib and atuveciclib treatments. Protein levels of cPARP, a marker for apoptosis, were 3- to 5-fold higher at peak occurring 24 hours after treatment washout in SU-DHL-10 cells treated with enitociclib or atuveciclib in comparison with KB-0742, which had no effect on cPARP (Fig. 2E). These results suggest that sustained *in vitro* control of Myc protein levels and activation of cell death in *MYC+* lymphoma cell line models are driven by enitociclib in a dose-dependent manner.

### Enitociclib Treatment Results in Complete Regression of *MYC-*overexpressing SU-DHL-10 Lymphoma Growth and Mechanism of Action Confirmed *In Vivo*

**FIGURE 3:**
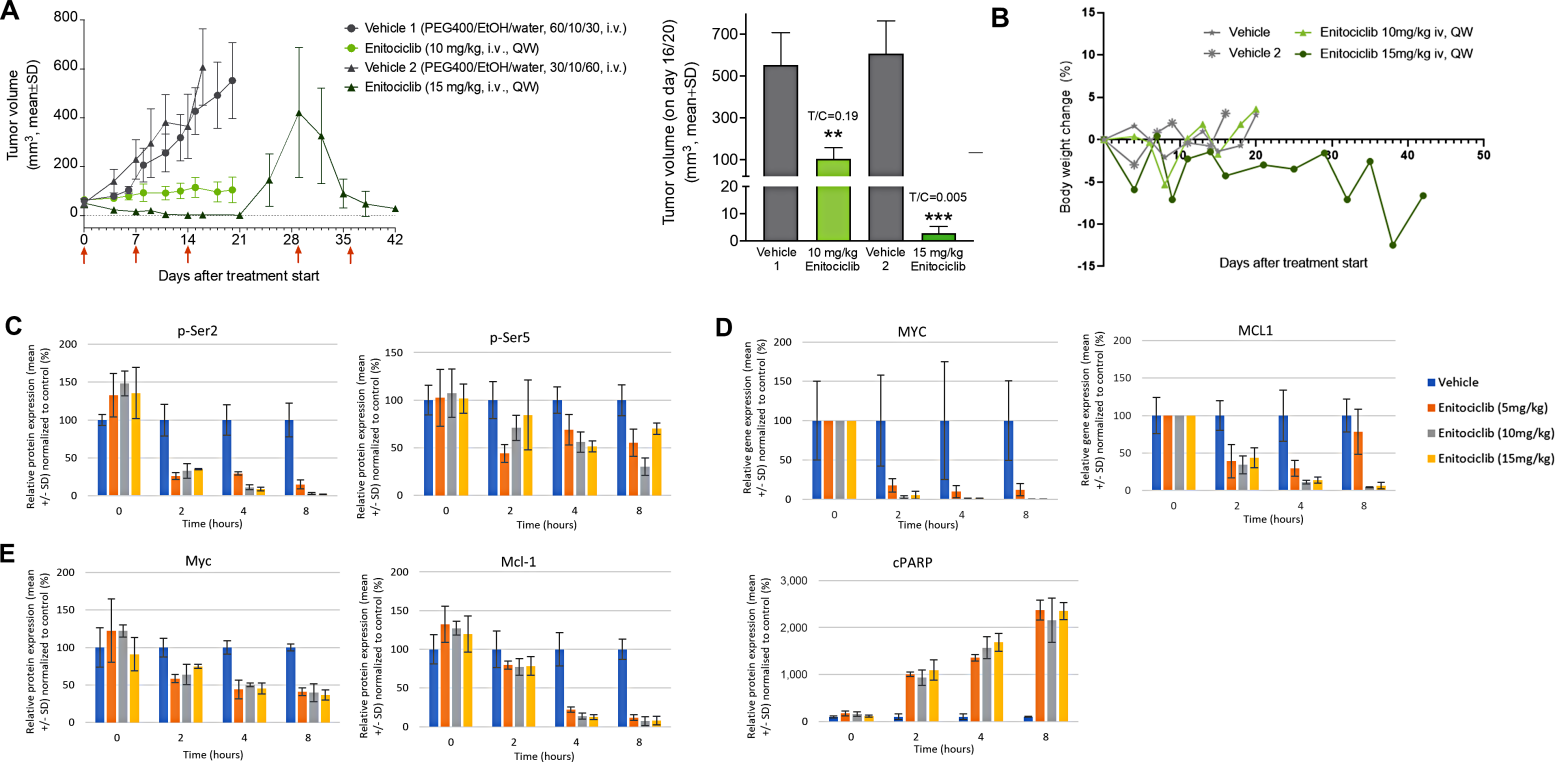
Enitociclib treatment results in complete regression of *MYC*-overexpressing SU-DHL-10 lymphoma growth and mechanism of action confirmed *in vivo*. **A,** Growth curves where red arrows indicate dosing days and tumor volumes of SU-DHL-10 tumors treated with either vehicle [30 or 60% PEG400, 10% ethanol (EtOH), water for infusion] or enitociclib at 10 or 15 mg/kg (once weekly, i.v., *n* = 12/group) for 3 weeks. On days 16 and 20, the T/C ratios for 10 and 15 mg/kg enitociclib were 0.19 and 0.005, respectively. Unpaired *t* test were performed using comparison with the corresponding vehicle treatment: **, *P* = 0.011; ***, *P* < 0.001. **B,** Body weight changes were observed after application of enitociclib followed by recovery before the next application. **C,** Phosphorylated RNA polymerase II protein levels at residue Ser2 (p-Ser2) and Ser5 (p-Ser5) were quantified MYC and MCL1 mRNA levels (**D**) assessed by qPCR and Myc, Mcl-1, cPARP protein levels **(E)** were quantified from *in vivo* xenograft of SU-DHL-10 DLBCL cells at predose and indicated times postdose *in vivo*. Error bars denote SD.

The antitumor efficacy of enitociclib was evaluated in the SU-DHL-10 *in vivo* xenograft mouse model. A dose-dependent antitumor efficacy was observed, resulting in SU-DHL-10 tumor growth control with 10 mg/kg enitociclib. Whereas 15 mg/kg enitociclib induced complete regression during the 3-week once weekly treatment period (Fig. 3A). On days 16 and 20 after treatment start, the treatment/control (T/C) values for 10 and 15 mg/kg enitociclib were 0.19 (*P* = 0.011) and 0.005 (*P* < 0.001), respectively (Fig. 3A). Subsequently, tumors in mice treated with 15 mg/kg enitociclib were allowed to regrow until a new treatment cycle with enitociclib (15 mg/kg, once weekly) was introduced on study day 29 again leading to regression of tumors (Fig. 3A). Changes in body weights of treated mice were observed during the study where recovery is observed before the next weekly dose and the maximum body weight change observed was −12.5% at 15 mg/kg (Fig. 3B). The pharmacodynamic effect of enitociclib in the tumors of SU-DHL-10 *in vivo* xenograft mouse model was evaluated after a single dose of 5, 10, or 15 mg/kg. Treatment with 5 mg/kg enitociclib decreased the levels of p-Ser2 by greater than 50% for 8 hours; whereas the 10 and 15 mg/kg doses resulted in depletion of p-Ser2 in the same timeframe postdose. In contrast, p-Ser5 reductions at all doses were not robust (Fig. 3C). A dose-dependent depletion of *MYC* mRNA detected by qPCR was observed. At the 10 and 15 mg/kg doses, *MYC* mRNA is depleted by 4 hours postdose. Apoptotic regulator *MCL1* mRNA levels were also reduced by 4 hours postdose, and depletion was sustained for 8 hours postdose at the two higher dose levels. (Fig. 3D). Myc protein levels were reduced by 50% and Mcl-1 protein levels robustly decreased within 4 to 8 hours postdose. Activation of apoptosis was observed as measured by an increase in cleaved PARP (Fig. 3E).

### Enitociclib Treatment Confers a Robust Shift in Transcriptional Activity in *MYC*+ DLBCL Cell Lines

**FIGURE 4:**
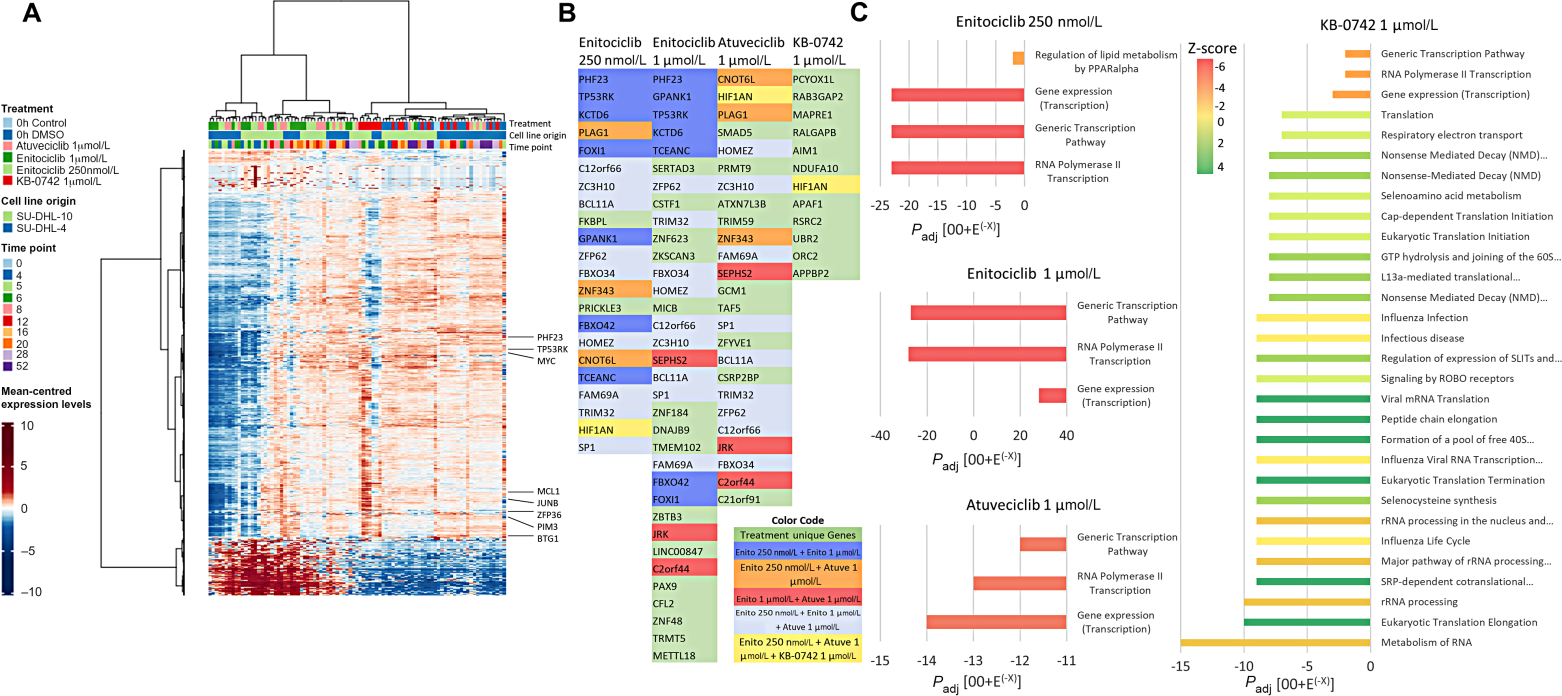
Enitociclib treatment confers a robust shift in transcriptional activity in *MYC*+ DLBCL cell lines. **A,** Comparison of significant DEGs (*P*_adj_ ≤ 0.05, fold change ≥2) after 4 hours pretreatment of DLBLC cell lines with three CDK9 inhibitors with unsupervised hierarchical clustering based on 250 nmol/L enitociclib treatment. Heat map showing gene intensity per sample relative to the average level across all samples. Individual genes are shown on the *Y* axis while samples are shown along the *X* axis. Red and blue cells correspond to higher and lower RNA-seq levels, respectively after 4 hours of treatment. **B,** List of top DEGs for each treatment ranked by *P*_adj_ (cutoff 10–7) with overlapping DEGs as indicated by color coding. **C,** Significant DEGs were analyzed for enrichment of Reactome pathway membership using a hypergeometric test by mapping genes to genes (if appropriate) for each treatment condition and for KB-0742 treated cells, the *P*_adj_ value cutoff for the pathway of 10–7 was selected for graphical representation because there were 182 significant pathways (30 shown). The three top pathways for enitociclib and atuveciclib are shown for KB-0742 but the *P*_adj_ values do not meet the cutoff but are included for comparative purposes.

Given that the P-TEFb complex regulates RNA polymerase II transcription via CDK9 kinase activity, the effects of three CDK9 inhibitors, enitociclib, atuveciclib, and KB-0742 on gene expression in SU-DHL-4– and SU-DHL-10–treated cell lines (Fig. 1B) was assessed by RNA-seq. According to PCA, the transcriptional profiles are most variable based on their cell line of origin and then the variance in the raw data is explained mostly by timepoint (Supplementary Fig. S1A). RNA-seq profiles from both cell lines were compared with each other for each treatment as well as compared with DMSO treatment and parental cell lines. A heat map of 1,491 significantly DEGs after 4 hours 250 nmol/L enitociclib treatment demonstrates gene intensity per sample relative to the average level across all samples for all treatments. Unsupervised clustering of samples demonstrates that enitociclib treatment (250 nm and 1 µmol/L) and 1 µmol/L atuveciclib cluster together while those samples treated with DMSO or 1 µmol/L KB-0742 cluster together. The majority of DEGs are downregulated (1,177/1,491; 78.94%) which drives the clustering of genes into two branches of the dendrogram (Fig. 4A). The top DEGs from RNA-seq analysis of samples from cell lines treated with each of the four treatments are listed in order of their *P*_adj_ < e-7 and unique or common DEGs are color coded (Fig. 4B), and fold change and *P* values are listed (Supplementary Table S3). The top DEGs in the cell lines treated with 250 nmol/L enitociclib were PHF23 (fold change −12.236; *P*_adj_ 1.14 e-12) and TP53RK (fold change −9.992; *P*_adj_ 7.76 e-8). Both PHF23 and TP53RK were statistically significantly downregulated with 1 µmol/L enitociclib but not with atuveciclib or KB-0742 treatment. A functional analysis of DEGs for the four treatments was undertaken to identify enriched pathways and describe the transcriptional changes due to CDK9 inhibition. With 250 nmol/L enitociclib treatment, which is at equivalent exposures to the 30 mg clinical dose, individual Reactome pathways were assessed for significant enrichment and four pathways were identified: regulation of lipid metabolism by PPAR alpha, gene expression (transcription), generic transcription pathway, RNA polymerase II transcription where the three latter pathways have *P*_adj_ values of e-23 with z-scores of −5. Three of the four identified pathways in enitociclib and atuveciclib 1 µmol/L treatments were the same, where generic transcription, RNA polymerase II transcription and gene expression (Transcription) pathways were statistically significant with a *P*_adj_ < e-7 (Fig. 4C). These three Reactome pathways were not the top pathways identified in the KB-0742 treatment condition, but they were statistically significant with *P*_adj_ between e-3 and e-2. These data confirm that the mechanism of action of CDK9 inhibition is to regulate RNA polymerase II mediated transcription as well as other generic transcriptional pathways.

### Baseline Characteristics, Enitociclib Response, and Pharmacokinetic Properties of Patients with DH-DLBCL and Other MYC+ NHL (*n* = 15) Treated with 30 mg Enitociclib i.v. Once Weekly

As reported previously, complete and durable molecular response (CR) was observed in 2 of 7 patients with DH-DLBCL treated with 30 mg enitociclib i.v. once weekly in a phase I clinical trial (29). In further expansion cohorts of the same trial, an additional 9 patients with DH-DLBCL or other MYC+ NHL were recruited (ClinicalTrials.gov Identifier: NCT02635672). Collectively, 15 of 16 patients had longitudinal blood collections taken at predose and postdose to evaluate the pharmacodynamic effect of enitociclib (30 mg, i.v. once weekly). Demographics of the 15 patient cohort consists of 10 (66.7%) with DH-DLBCL and 5 others with MYC+ NHL, 9 (60%) with ≥ 3 prior therapies, 4 (26.7%) refractory to last treatment, 6 (40%) with bulky disease > 7.5 cm, and 10 (66.7%) Ann Arbor staging at study entry stage IV (Supplementary Table S4). Clinical response to enitociclib for DH-DLBCL [10; 2 CR, 3 progressive disease (PD), 5 not evaluable (NE)], triple hit-DLBCL (1 NE), mantle cell lymphoma (1 NE), DLBCL-Richter (1 NE), Burkitt lymphoma (1 PD), transformed follicular lymphoma [1 stable disease (SD)] where treatment duration of 2 metabolic CR patients was 194 and 122 weeks, respectively and for the transformed follicular patient, SD was 25.6 weeks at 03DEC2022 data extract and remains on treatment at time of writing. The observed pharmacokinetic properties of enitociclib for patients with DH-DLBCL who achieved a metabolic CR (*n* = 2) is within the range of other patients with DH-DLBCL whose best overall response was NE or PD. Similarly, the pharamcokinetic properties of patients with other MYC+ NHL were comparable with what was observed for the patients with DH-DLBCL (Table 1).

**TABLE 1:** Response and pharmacokinetics properties of patients with DH-DLBCL and other patients with MYC+ NHL (*n* = 15) treated with 30 mg enitociclib i.v. once weekly

			Single dose						Multiple dose				Best	Treatment
			*C*_max_	t_max_	AUC_0-t_	CL	Vss	t_1/2_	*C*_max_	t_max_	AUC_0-t_	t_1/2_	Overall	Duration
Subject	Indication	Cohort	µg/L	h	µg^a^h/L	L/h	L	h	µg/L	h	µg^a^h/L	h	response	(weeks)
1	Double Hit DLBCL	DH-DLBCL	441	0.583	1,350	22.3	74.2	5.68	—	—	—	—	Not evaluable	1.14
2	Double Hit DLBCL	DH-DLBCL	622	0.250	3,790	7.44	59.6	6.15	776	0.283	3,890	7.03	Progressive disease	3.00
3	Double Hit DLBCL	DH-DLBCL	716	0.500	2,140	14	49.7	2.73	592^b^	0.483^b^	1,670^b^	2.53^b^	Complete remission	194
4	Double Hit DLBCL	DH-DLBCL	561	0.583	3450	—	—	—	524	0.617	3,020	4.27	Progressive disease	3.15
5	Double Hit DLBCL	DH-DLBCL	826	0.533	3,410	8.6	51.5	4.45	894	0.583	3,190	4.08	Not evaluable	2.14
6	Double Hit DLBCL	DH-DLBCL	1,020	0.500	3,220	9.24	42.2	3.32	847	0.617	2,490	4.54	Complete remission	122
7	Double Hit DLBCL	DH-DLBCL	1,010	0.517	1,980	15.1	42.6	2.48	844	0.567	1,660	2.58	Not evaluable	3.29
8	Double Hit DLBCL	DH-DLBCL	474	0.500	2,260	5.87	78.6	9.41	783	0.500	3,720	7.21	Not evaluable	3.00
9^a^	Double Hit DLBCL	DH-DLBCL	448	0.670	3,110	9.19	71.7	5.41	410	0.500	2,760	5.48	Progressive disease	5.14
10	Double Hit DLBCL	DH-DLBCL	558	0.500	3,370	8.37	71.3	6.02	581	0.500	3,090	7.24	Not evaluable	2.14
11	Triple Hit DLBCL	Other NHL	386	0.500	2,030	14.2	97.9	5.22	599	0.500	2,080	4.36	Not evaluable	4.00
12	Transformed follicular lymphoma	Other NHL	517	0.500	3,990	6.32	80.3	9.15	699	0.5	3,750	8.81	Stable disease	25.6
13	Burkitt's lymphoma	Other NHL	392	0.670	3,230	7.87	98.7	8.93	—	—	—	—	Progressive disease	5.14
14	Mantle cell lymphoma	Other NHL	2,150	0.500	4,260	6.96	39.2	3.88	—	—	—	—	Not evaluable	6.00
15	DLBCL – (Richters)	Other NHL	506	0.500	1,930	8.68	82.4	6.78	—	—	—	—	Not evaluable	1.14
	Mean		709	0.520	2,900	10.3	67.1	5.69	696	0.517	2,965	5.56		
	SD		448	0.096	879	4.5	19.9	2.28	161	0.096	736	1.93		

^a^0.5 hour (single dose) concentration of 12,200 ng/mL excluded from analysis.

^b^Dose was lowered from 30 to 22.5 mg (pharmacokinetic data were not included in average).

### Downregulation of *MYC* and *MCL1* is Detected in the Whole Blood of Patients with Enitociclib-treated DH-DLBCL as well as Other MYC+ NHL

To understand whether the downregulation of MYC and MCL1 *in vitro* translates to patients, the maximum extent of inhibition for *MYC* mRNA after the first dose of 30 mg enitociclib on C1D1 in patients with DH-DLBCL (*n* = 10, black lines) and other MYC+ NHL (*n* = 5, colored lines) whole blood was 89.6% (range, 73.6%–94.8%) and 82.3% (range, 69.9%–90.5%), respectively where maximal inhibition occurred 1–2 hours postdose. On C1D1, maximal *MCL1* mRNA inhibition occurred at 1–2 hours postdose except for patient with the transformed follicular lymphoma, which was observed at 8 hours postdose. The maximal extent of *MCL1* mRNA downregulation in patients with DH-DLBCL and other MYC+ NHL was 49.28% (range, 31%–52.4%) and 44.54% (range, 22.5%–48.2%), respectively (Supplementary Fig. S2A). The pharmacodynamic effect was reproduced on C1D15 after the third weekly dose of 30 mg enitociclib where *MYC* mRNA downregulation in patients with DH-DLBCL (*n* = 9, black lines) and on other MYC+ NHL (*n* = 3, colored lines) was 86.71% (range, 96.85%–50%) and 71.08% (range, 53.7%–86.9%), respectively. Downregulation of *MCL1* mRNA in patients with DH-DLBCL and other MYC+ NHL on C1D15 was 45.81% (range, 30%–55.5%) and 18.27% (range, 0%–43.1%), respectively. The modeled pharmacokinetic-pharmacodynamic relationship for *MYC* and *MCL1* mRNA inhibition following C1D1 (Supplementary Fig. S2C), and the associated model fitted pharmacokinetics (Supplementary Fig. S2D) show that no differences were observed in pharmacokinetic and pharmacodynamic response between patients with DH-DLBCL and other MYC+ NHL. The model estimated unbound enitociclib IC_50_’s for *MYC* and *MCL1* mRNA are in the range of 17.4–22.0 nmol/L and 61.2–69.1 nmol/L, respectively and did not show any time dependency (Supplementary Table S5). These IC_50_ estimates are comparable with mean unbound IC_50_ for cytotoxicity estimated for lymphoma cell lines (∼67 nmol/L; media f_u_ = 66.6%) in Supplementary Table S1.

### Comparison of DEGs Identified in MYC+ DLBCL Cell Lines to Blood Samples from Patients with Enitociclib-treated MYC+ NHL

**FIGURE 5:**
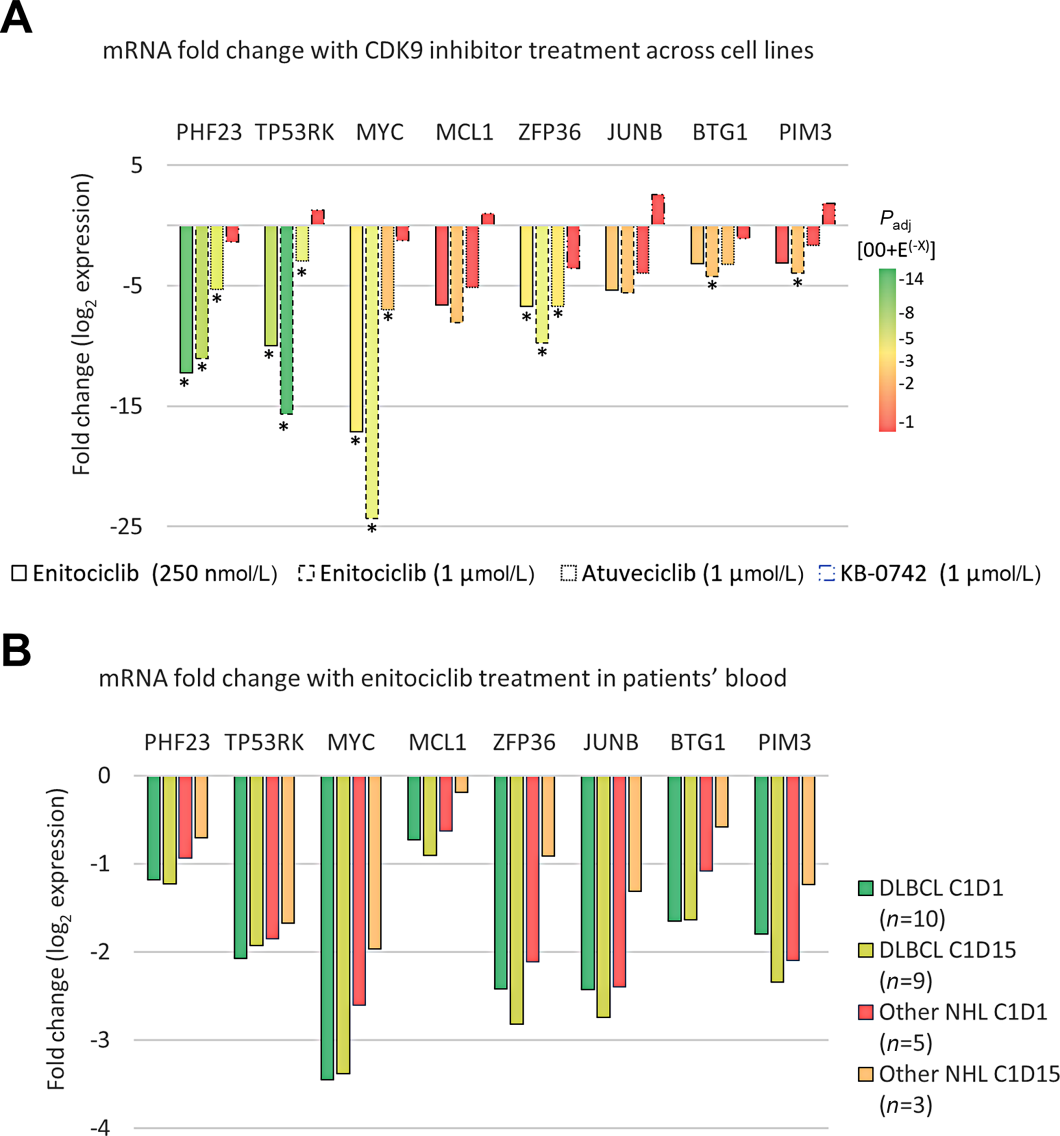
Comparison of DEGs identified in MYC+ DLBCL cell lines to blood samples from enitociclib-treated patienst with MYC+ NHL. **A,** Comparison of significant DEGs (*P*_adj_ ≤ 0.05, fold change ≥2) after at 4 hours pretreatment with 250 nmol/L enitociclib. Heat map showing gene intensity per sample relative to the average level across all samples. Individual genes are shown on the *Y* axis while samples are shown along the *X* axis. Red and blue cells correspond to higher and lower RNA-seq levels, respectively after 4 hours of treatment. **B,** Transcription factors MYC, JUNB, ZFP36 mRNA have statistically significant fold change downregulation with enitociclib treatment in DLBCL cell lines SU-DHL-4 and SU-DHL-10 where other 4 hours treatments do not. PHF23 and TP53RK were the most significantly DEGs with enitociclib treatment at both concentrations. * denotes *P*_adj_ ≤ 0.05 and fold change ≥2.

Because *MYC* and *MCL1* mRNA downregulation was observed in cell lines by qPCR *in vitro* (Fig. 2A and B) and *in vivo* (Fig. 3D), comparison with RNA-seq *in vitro* was evaluated. *MYC* mRNA is statistically significantly downregulated with enitociclib and atuveciclib but was not statistically significant with KB-0472 treatment (Fig. 5A). *MCL1* downregulation is observed but not statistically significant for enitociclib and atuveciclib treatment, whereas *MCL1* mRNA levels are upregulated with KB-0742 treatment. Of note, there are three *MCL1* mRNA splicing variants, of which only one is detected by the qPCR method, but all three are averaged by RNA-seq. RNA-seq analysis from other studies with enitociclib (39) and AZD4573 (40) treatment of CLL and DLBCL cell lines respectively have shown that ZPF36, JUNB, BTG1, and PIM3 are key DEGs with CDK9 inhibition. Herein we show that ZPF36 is significantly downmodulated with enitociclib and atuveciclib treatment and that only 1 µmol/L enitociclib provides statistically significant downregulation of BTG1 and PIM3 (Fig. 5A). DEG analysis by RNA-seq of DH-DLBCL (*n* = 10) and other MYC+ NHL (*n* = 5) patient blood samples predose and at multiple timepoints after 30 mg intravenous infusion of enitociclib on C1D1 and C1D15 was performed. PCA demonstrates a robust time-dependent shift in gene expression in patients treated with enitociclib (Supplementary Fig. S1B). The top two DEGs from the 250 nmol/L enitociclib 4 hours *in vitro* treatment (Fig. 5A, green bars) are confirmed to be reproducibly downregulated after C1D1 and C1D15 in blood samples of patients with DH-DLBCL and other MYC+ NHL at approximately –1- and –2-fold change from enitociclib predose for PHF23 and TP53RK mRNAs, respectively (Fig. 5B). Known CDK9 target genes from the literature MYC, MCL1, ZFP36, JUNB, BTG1, and PIM3 are shown to compare the extent of downregulation of novel targets PHF23 and TP53RK. Downregulation of transcription factors *ZFP36* and *JUNB* mRNA levels were > −2 in patients with DH-DLBCL after C1D1 and C1D15 and were approximately −2-fold change after C1D1 and decreased toward −1 after C1D15 in the blood of patients with other MYC+ NHL (*n* = 3). Similarly, *BTG1* and *PIM3* were also confirmed to be downregulated in the blood of patients treated with enitociclib whereas *MCL1* mRNA fold change was the least robust (Fig. 5B). In support of the mechanism of action elucidated herein, *MYC* fold change downregulation in the blood of patients treated with enitociclib was the most robust finding in this group of genes. The mean fold change mRNA levels of *MYC* in patients with DH-DLBCL and other MYC+ NHL were −3.45 (SD 0.68) and −2.61 (SD 0.54), respectively after C1D1 dose; and after C1D15 dose, *MYC* mRNA fold change was −3.38 (SD 1.07) and −1.97 (SD 0.75) for DH-DLBLC and other MYC+ NHL, respectively. These data confirm that robust *MYC* mRNA downregulation is consistent across *in vitro*, *in vivo* and blood samples from patients with NHL treated with enitociclib. The maximal DEG inhibition with enitociclib treatment demonstrates that MYC is the most robust, but the time course for maximal DEG inhibition suggests a MYC-independent mechanism where novel DEGs identified here PHF23 and TP53RK have maximal inhibition first, followed by known CDK9 targets ZFP36 and PIM3 maximal inhibition, then MYC, MCL1, JUNB, and BTG1 (Supplementary Table S6).

## Discussion

Here we investigated the mechanism of action of enitociclib *in vitro*, *in vivo* and in blood samples from patients with NHL. Enitociclib was previously shown to drive durable single-agent complete metabolic responses in patients with DH-DLBCL in a phase I trial (29). We show that CDK9 inhibition at clinically relevant concentrations (250 nmol/L enitociclib), results in inhibition of RNA polymerase II Ser2 phosphorylation, the direct substrate of CDK9. Inhibition of Ser2 phosphorylation is confirmed *in vivo* and is demonstrated to be selective because Ser5 phosphorylation, a residue known to be regulated by CDK7 phosphorylation (41), is not inhibited confirming that enitociclib is a selective CDK9 inhibitor *in vivo*. Because RNA polymerase II Ser2 phosphorylation triggers mRNA elongation (42), we examined changes in *MYC* and *MCL1* mRNA levels and demonstrate an initial decrease with all three CDK9 inhibitors tested *in vitro*; however, only atuveciclib and enitociclib were able to drive the complete depletion of *MYC* and *MCL1* mRNA transcript levels after a short 4-hour pretreatment then washout. This pharmacodynamic effect in mRNA levels mirrors the p-Ser2 RNA polymerase II dynamics observed in both cell lines. The magnitude of the enitociclib pharmacodynamic effect is demonstrated by a sustained reduction and near clearance of Myc and Mcl-1 proteins in *MYC-*expressing lymphoma cell lines *in vitro* and *in vivo*. Furthermore, we demonstrate that treatment with enitociclib triggers apoptosis, which leads to tumor regression because increases in caspase-3 cleavage were observed as early as 8 hours postdose and dose-dependent antitumor efficacy and tumor-outgrowth control in the SU-DHL-10 *in vivo* model.

These results demonstrate that intermittent weekly dosing with enitociclib regulates multiple parallel pathways including oncogenic Myc and antiapoptotic Mcl-1, previously described to overcome the oncogene addiction through differential signal attenuation where prosurvival signals dissipate quickly on oncoprotein inactivation whereas proapoptotic signals linger sufficiently long to commit the cell to an apoptotic death (33, 34). These preclinical findings were translated into the clinical setting where we show that *MYC* and *MCL1* mRNA levels are downregulated in the blood of DH-DLBCL but that also this mechanism of action is confirmed in patients with other MYC+ NHL across various indications and not limited to DH-DLBCL. Although the extent or duration of downregulation does not correlate with enitociclib efficacy, the unbound IC_50_ of both *MYC* and *MCL1* mRNA are both clinically achievable with the 30 mg dose, even though the *MCL1* IC_50_ is equivalent to the *C*_max_ which is demonstrated by a 55.5% maximal downregulation of *MCL1 mRNA* level in blood of patients postdose. There are limitations to this study, namely the small patient sample size tested and the fact that mRNA levels were measured only from the blood of patients because tumor tissue was not available. A comparison of the pharmacodynamic effect in both blood and tumor tissue samples would provide direct evidence of target knockdown in tumor tissue, but this phase I clinical trial did not mandate pretreatment and posttreatment tumor biopsies.

To understand the transcriptional impact of CDK9 inhibition beyond known targets such as MYC and MCL1, an unbiased transcriptomic analysis has identified DEGs that may be novel CDK9 gene targets in MYC+ DLBCL cell lines. Treatment with enitociclib using clinically relevant conditions has the expected transcriptional footprint for an inhibitor of RNA polymerase II where 78.94% DEGs are downregulated. In addition to the downregulation of MYC, other genes have been identified as being downregulated by CDK9 inhibition and this observation has been shown in some cases to precede MYC maximal downregulation, as is the case for the top two DEGs in the unbiased analysis, PHF23 and TP53RK. Pathway analysis revealed that RNA transcription pathways are driving the DEGs identified, which is fitting with the proposed mechanism of action. For relative comparison, MYC does not rank in the top DEGs of this study, but the extent of downregulation (−17.108-fold change) is greater than the novel DEGs identified further strengthening the utility of selective CDK9 inhibitors for targeting MYC+ lymphomas.

In support of the transcriptional shift observed, other transcription factors known to be downregulated by CDK9 inhibition were evaluated in the RNA-seq dataset. JUNB, ZFP36, and BTG1 were identified in an RNA-seq analysis of CLL cells treated with enitociclib *in vitro* (39). We demonstrate that in MYC+ DLBCL cell lines as well as in the blood of patients with DH-DLBCL and other MYC+ NHL, these three transcription factors are also downregulated. Furthermore, in DLBCL cell lines *in vitro*, PIM3 was identified as a key mediator of epigenetic resistance to another pan-CDK inhibitor, AZD4573 (40). We demonstrate that PIM3 was also downregulated by enitociclib treatment in DLBCL cell lines *in vitro* and in the blood of patients with DH-DLBCL and other MYC+ NHL. The authors demonstrated that PIM2/3, but not PIM1 inhibition could reverse the resistance (40) yet our data might suggest that rather than a combination of AZD4573 with pan-PIM inhibitor, single agent enitociclib could prevent or treat emergent resistance due to PIM3 overexpression, but confirmation in larger datasets would be required as well as supportive data from corresponding tumor tissue to match observations in blood samples. We further identify two novel targets in this study by RNA-seq of MYC+ DLBCL cell lines treated with 250 nmol/L enitociclib. PHF23 and TP53RK are downregulated and are the two top statistically significant DEGs with robust –10- to –12-fold change in expression postdose (*P*_adj_ e-12 and e-8), respectively. Histone H3 lysine methylation is a critical component in regulating gene expression, epigenetic states, and cellular identities and is interpreted by conserved modules including plant homeodomain (PHD) fingers of which PHD containing proteins have been deregulated in many types of cancers (43) including PHF23 which forms fusion oncoprotein with NUP98, which is associated with an aggressive form of AML (44). PHF23 is a negative regulator of autophagy (45) and perhaps explains escape from cell death. The fusion drives overexpression, therefore, downregulation with enitociclib might allow autophagy and provide a therapeutic opportunity for NUP98-PHF23–driven AML that has a poor survival rate. TP53RK has been linked with colorectal cancer metastasis (46) and identified as a target for multiple myeloma (47) and enitociclib may provide an opportunity to target TP53RK. Both PHF23 and TP53RK mRNA downregulation was confirmed by RNA-seq analysis in the blood of enitociclib-treated patients and further studies are warranted to uncover the utility of enitociclib to downregulate these potential cancer targets.

## Conclusion

This study demonstrates the biologic advantage of a selective CDK9 inhibitor for the treatment of *MYC*+ lymphoma preclinical models which is confirmed by analysis of blood samples from patients treated with 30 mg enitociclib i.v. once weekly. Robust transcriptomic down regulation of transcription factors MYC, JUNB, ZFP36, BTG1 as well as novel targets PHF23 and TP53RK was observed with weekly enitociclib dosing while providing a differential decay of prosurvival and antiapoptotic signals. Taken together, our data demonstrate that enitociclib treatment enables durable downregulation of oncogenic protein levels without continuous dosing, resulting in a robust transcriptional shift promoting cancer cell apoptosis and antitumor efficacy. Currently, enitociclib is being evaluated in patients diagnosed with DH-DLBCL and other relapsed/refractory lymphoid malignancies to determine safety and efficacy of enitociclib treatment given as monotherapy or in combination (ClinicalTrials.gov Identifiers: NCT04978779 and NCT05371054).

## Supplementary Material

Figure S1 Supplementary Figure 1 shows the principal component analysis of RNA sequencing of either SU-DHL-4 and SU-DHL-10 cell lines as well as that from RNA sequencing of patient blood samples.

Figure S2 Supplementary Figure 2: Downregulation of MYC and MCL1 is detected in the whole blood of Enitociclib-treated DH-DLBCL as well as other MYC+ NHL patients

Table S1 Supplementary Table 1 shows the enitociclib toxicity in a panel of lymphoma cell lines ranked by IC50

Table S2 Supplementary Table 2 contains the statistical analysis for pSer2 downregulation in SU-DHL-4 and SU-DHL-10 cell lines across CDK9 inhibitor treatments.

Table S3 Supplementary Table 3 contains the top DEGS from an unbiased analysis of RNAseq from MYC+ DLBCL cell lines treated with CDK9 inhibitors.

Table S4 Supplementary Table 4 shows the baseline characteristics of DH-DLBCL and other MYC+ NHL Patients (n=15) treated with 30 mg enitociclib i.v. QW from whom samples were collected for discovery of DEGs.

Table S5 Supplementary Table 5 shows estimated pharmacodynamic parameters for MYC and MCL1 mRNA in blood following enitociclib treatment. ​

Table S6 Supplementary Table 6 captures the maximal downregulation timepoint for novel and known DEGs post enitociclb treatment in MYC+ DLBCL patients.
