# Supplementary figures and images for: Enitociclib, a Selective CDK9 Inhibitor, Induces Complete Regression of MYC+ Lymphoma by Downregulation of RNA Polymerase II Mediated Transcription
Source: Cancer Res Commun. 2023 Nov 9;3(11):2268–79. doi: 10.1158/2767-9764.CRC-23-0219 (PMC10634346; doi:10.1158/2767-9764.CRC-23-0219)

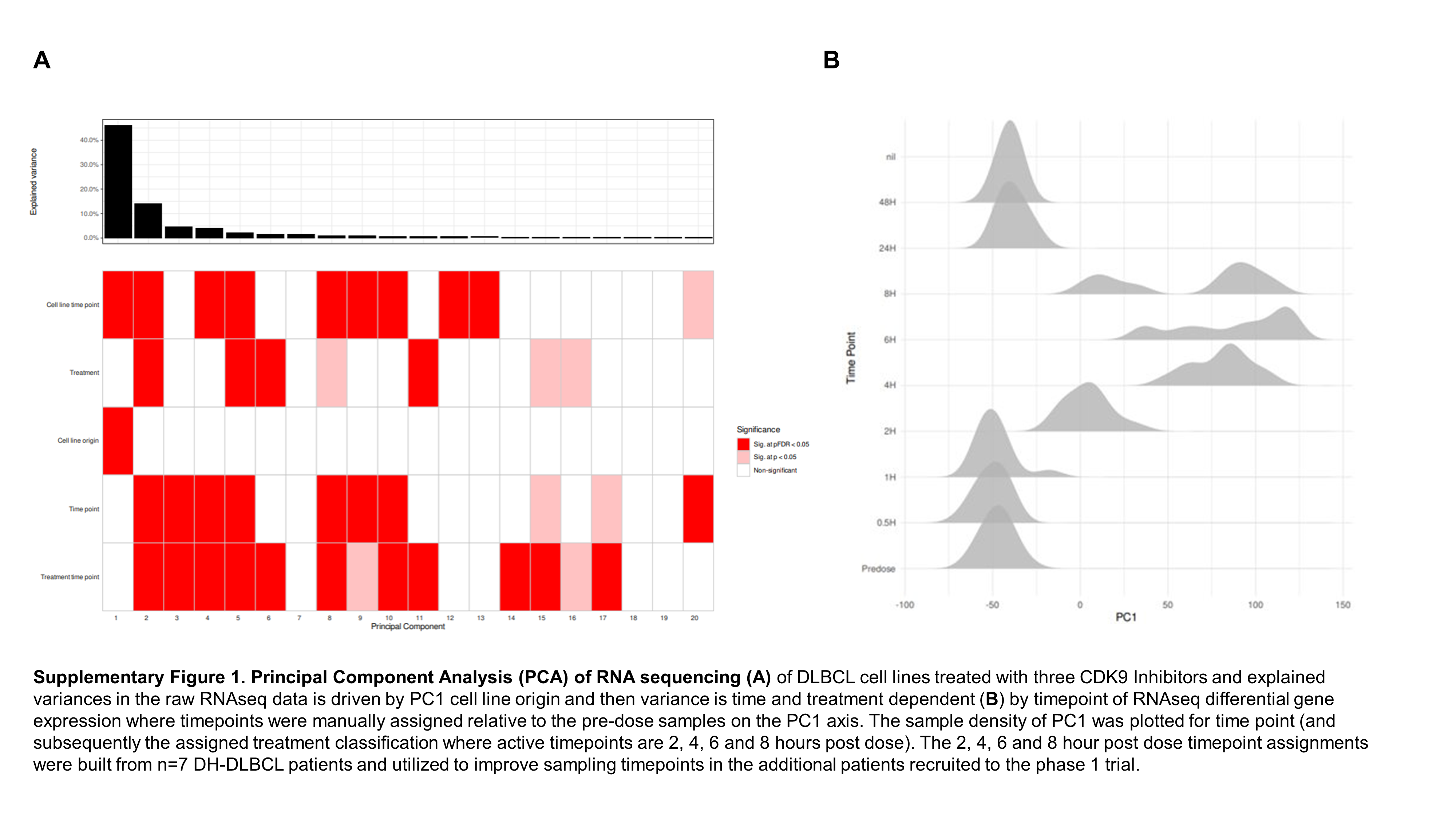

Supplement: Figure S1 — Supplementary Figure 1 shows the principal component analysis of RNA sequencing of either SU-DHL-4 and SU-DHL-10 cell lines as well as that from RNA sequencing of patient blood samples. [file crc-23-0219-s01.png]

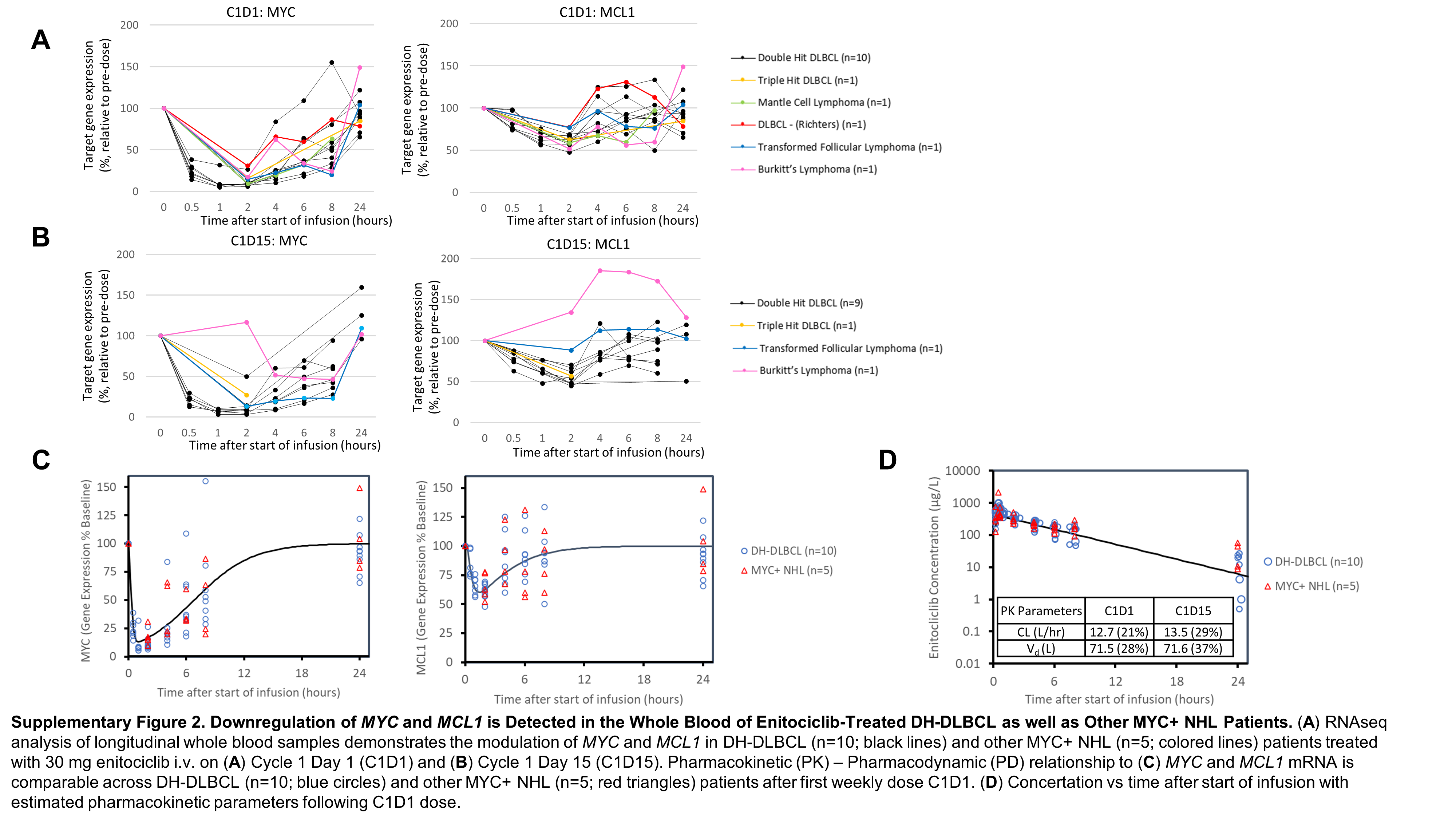

Supplement: Figure S2 — Supplementary Figure 2: Downregulation of MYC and MCL1 is detected in the whole blood of Enitociclib-treated DH-DLBCL as well as other MYC+ NHL patients [file crc-23-0219-s02.png]

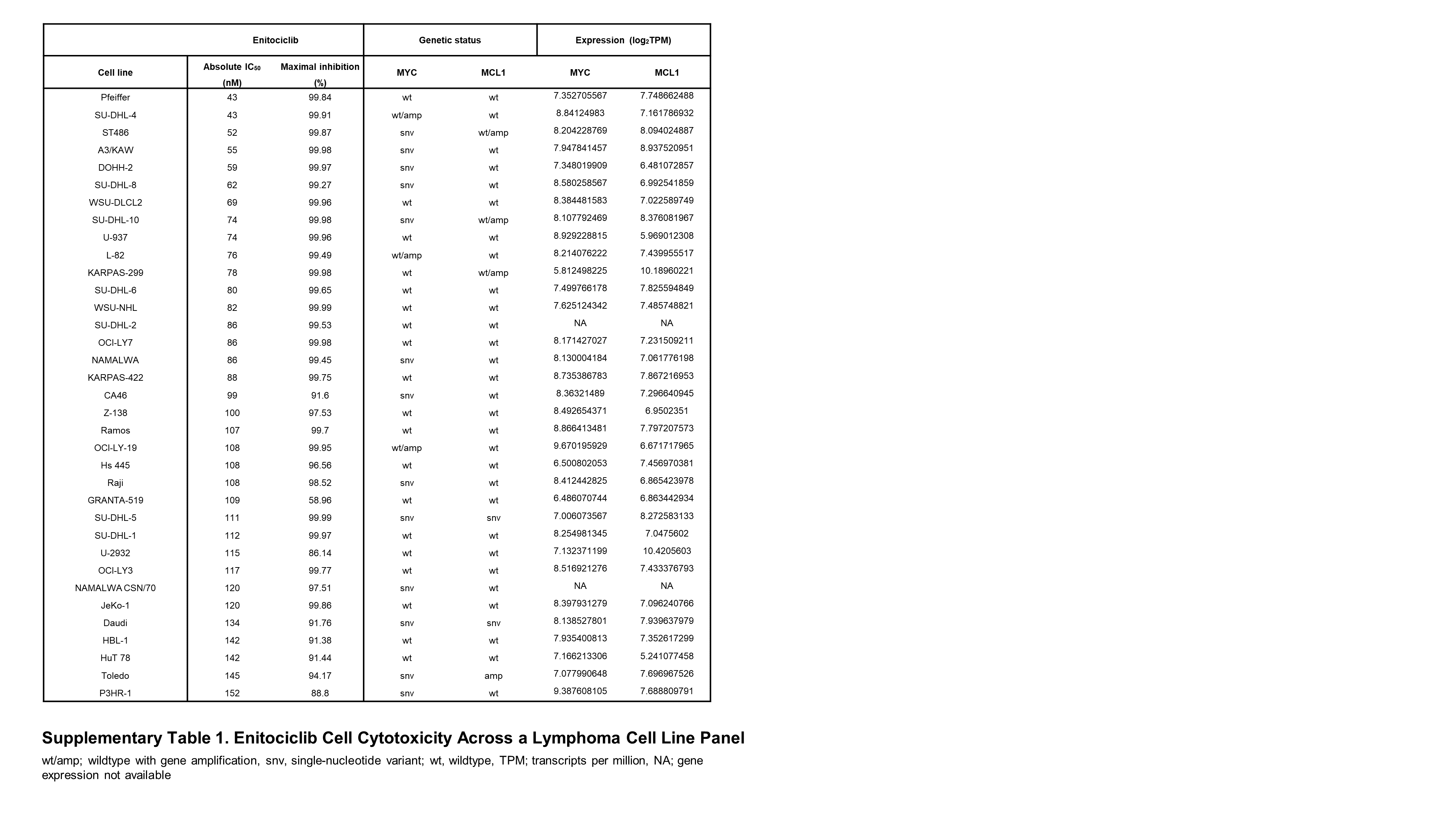

Supplement: Table S1 — Supplementary Table 1 shows the enitociclib toxicity in a panel of lymphoma cell lines ranked by IC50 [file crc-23-0219-s03.png]

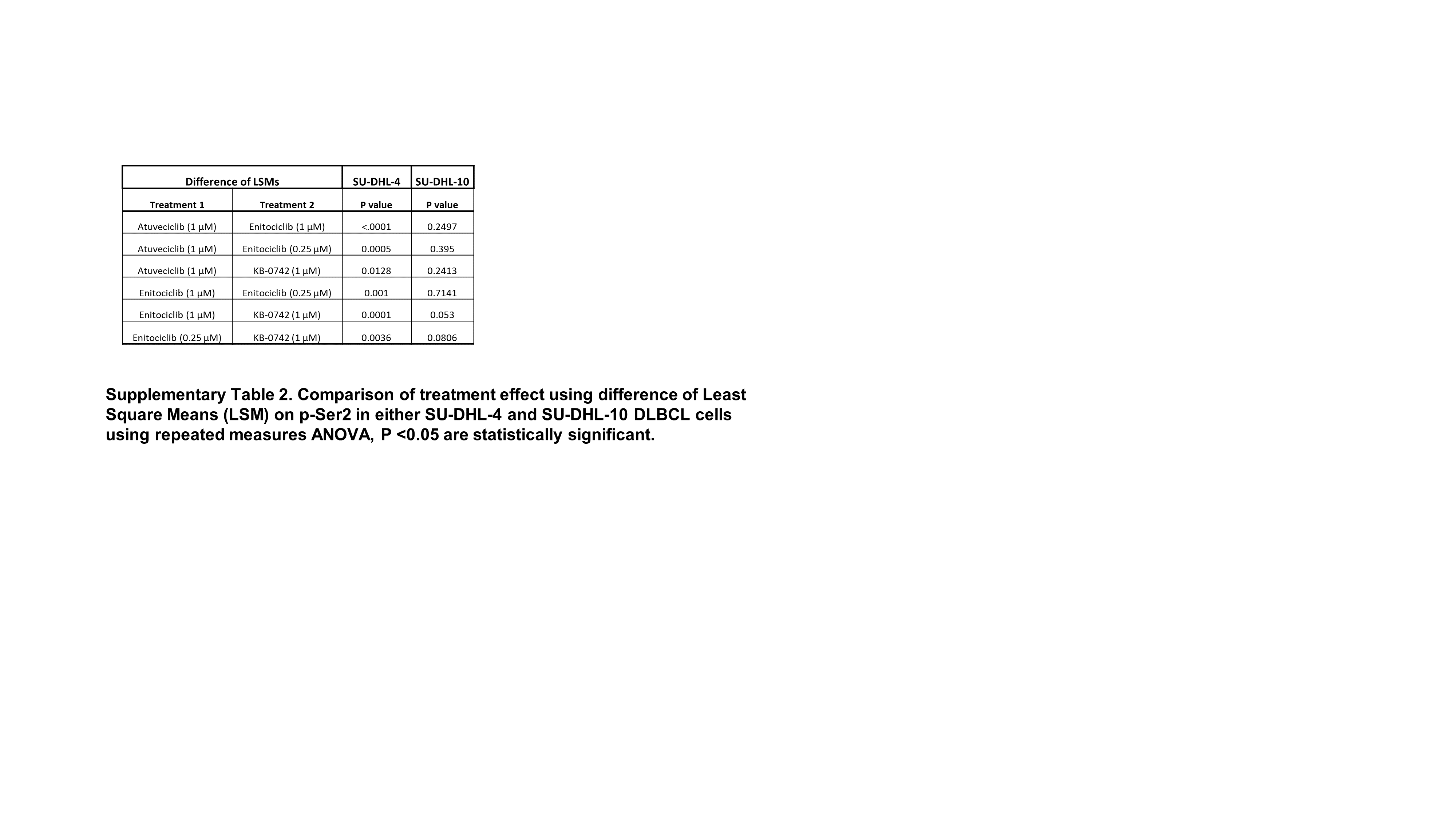

Supplement: Table S2 — Supplementary Table 2 contains the statistical analysis for pSer2 downregulation in SU-DHL-4 and SU-DHL-10 cell lines across CDK9 inhibitor treatments. [file crc-23-0219-s04.png]

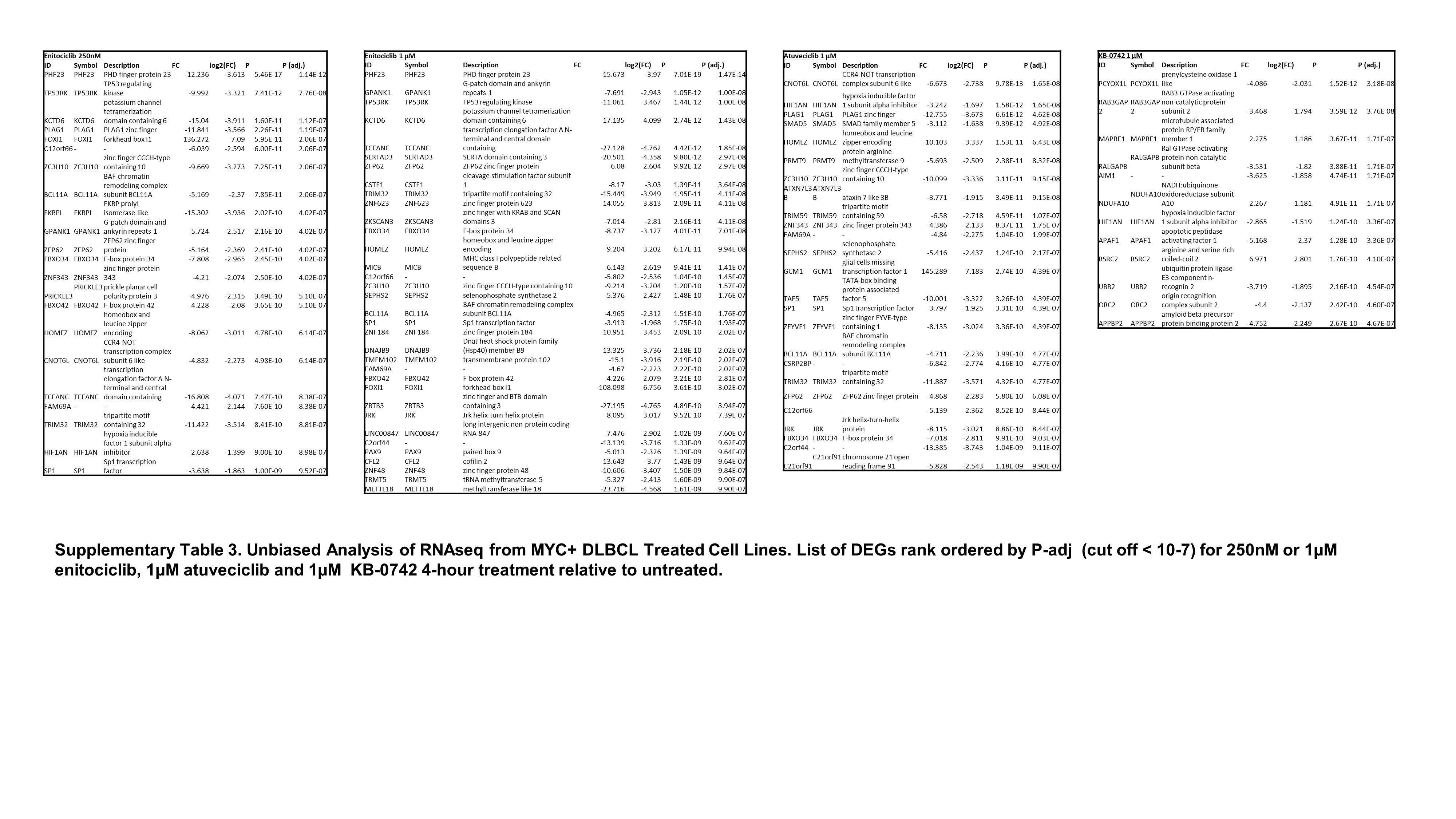

Supplement: Table S3 — Supplementary Table 3 contains the top DEGS from an unbiased analysis of RNAseq from MYC+ DLBCL cell lines treated with CDK9 inhibitors. [file crc-23-0219-s05.png]

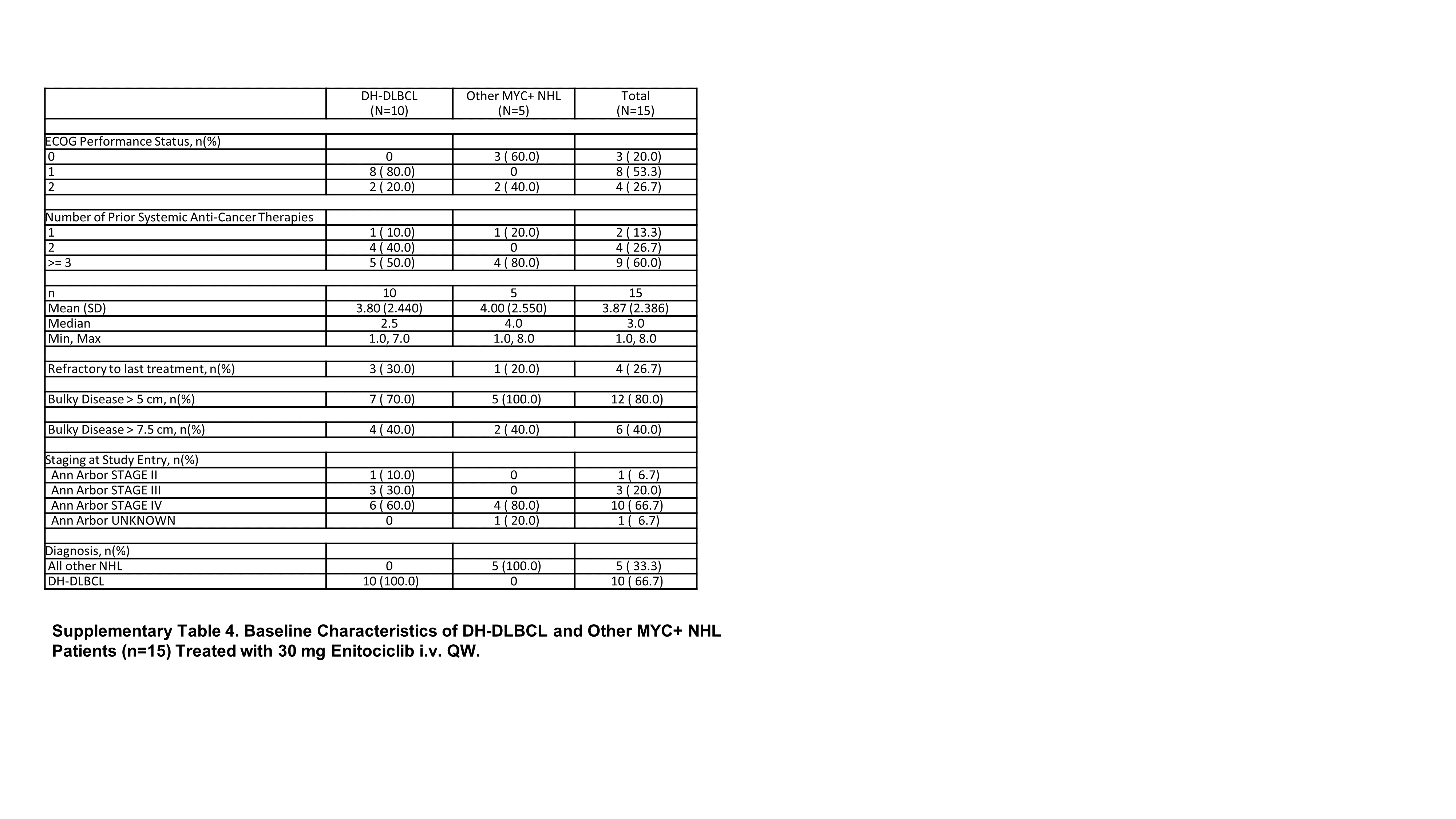

Supplement: Table S4 — Supplementary Table 4 shows the baseline characteristics of DH-DLBCL and other MYC+ NHL Patients (n=15) treated with 30 mg enitociclib i.v. QW from whom samples were collected for discovery of DEGs. [file crc-23-0219-s06.png]

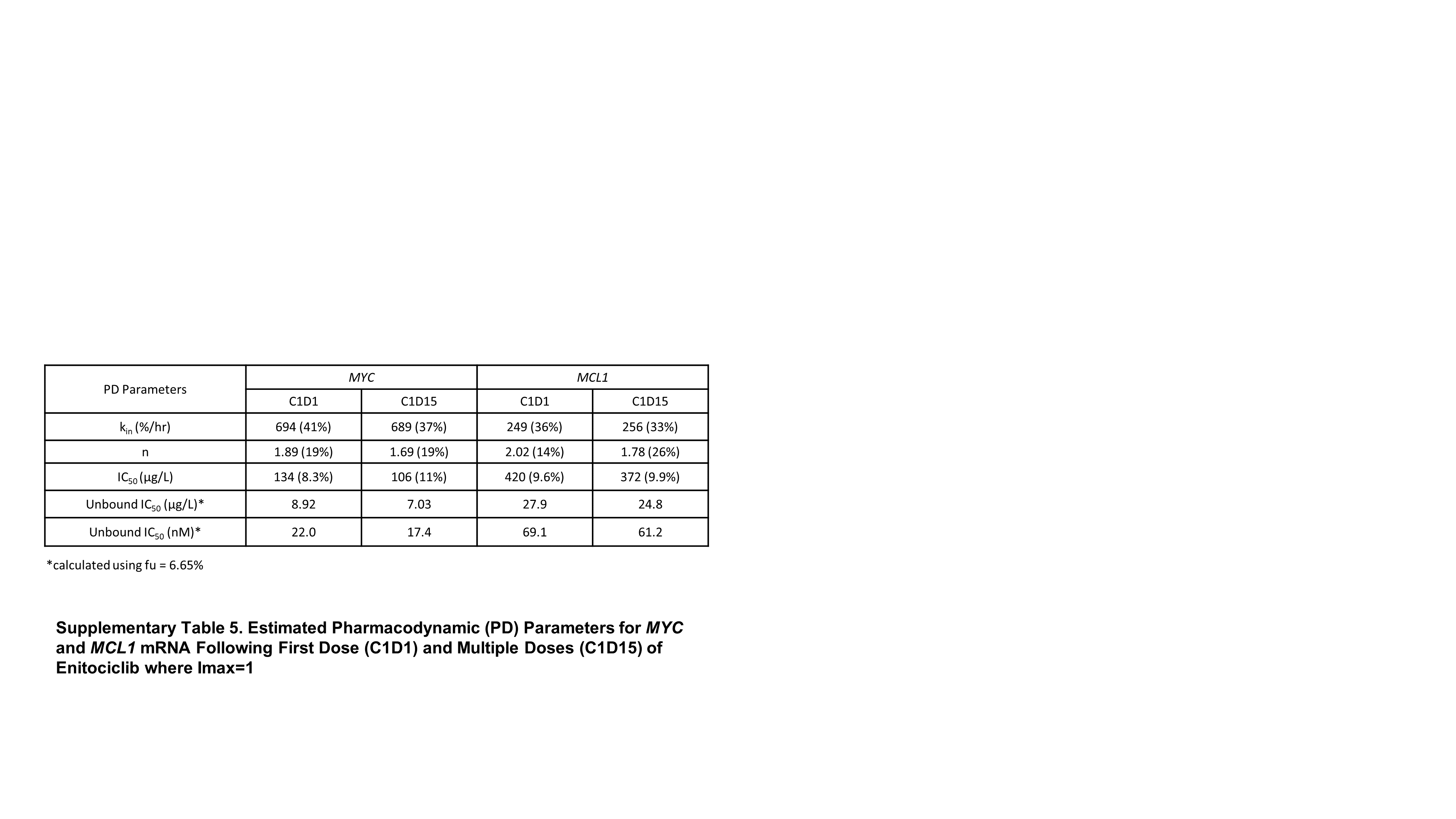

Supplement: Table S5 — Supplementary Table 5 shows estimated pharmacodynamic parameters for MYC and MCL1 mRNA in blood following enitociclib treatment. ​ [file crc-23-0219-s07.png]

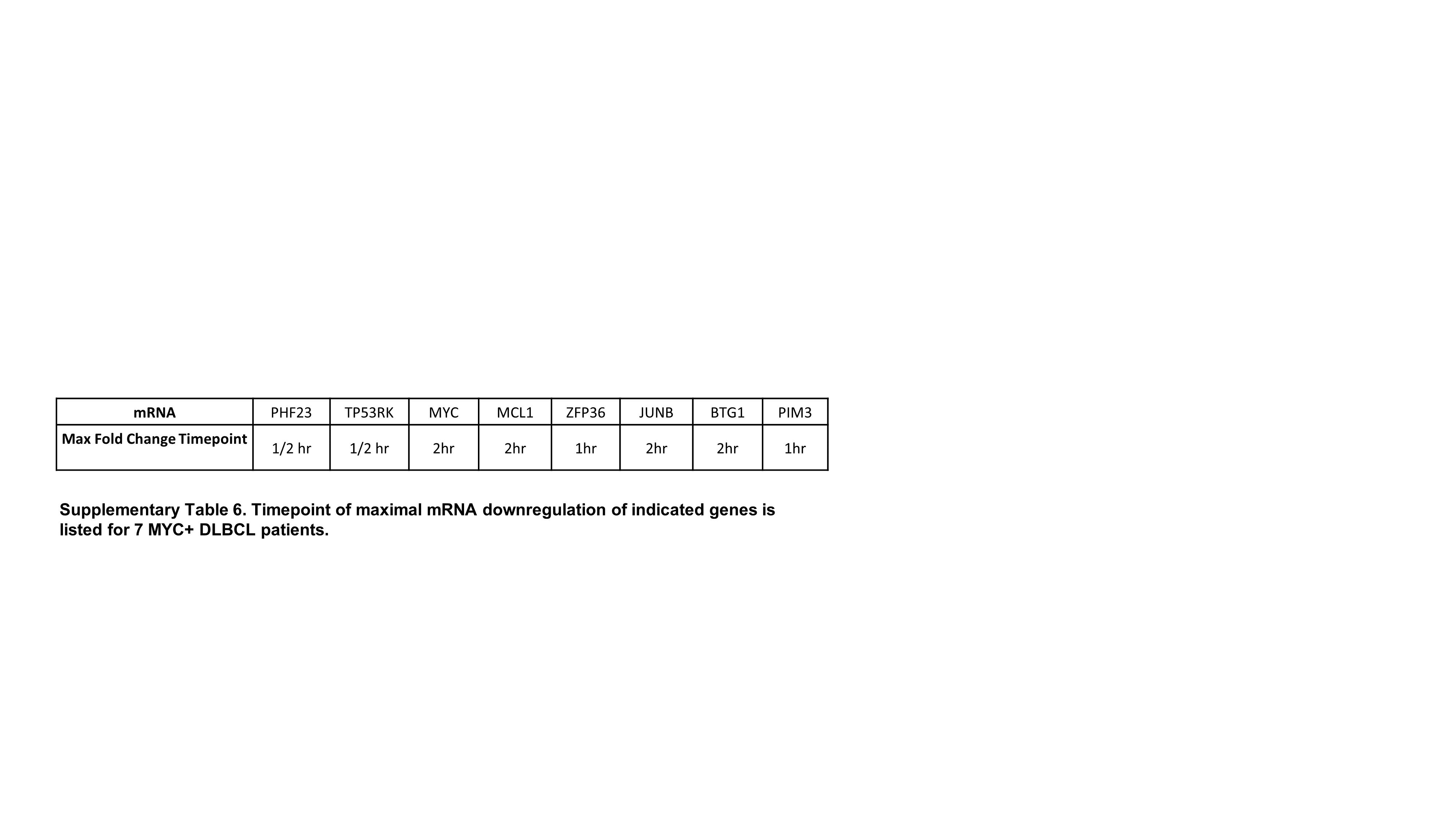

Supplement: Table S6 — Supplementary Table 6 captures the maximal downregulation timepoint for novel and known DEGs post enitociclb treatment in MYC+ DLBCL patients. [file crc-23-0219-s08.png]
